# Antioxidants and Bioactive Compounds in Licorice Root Extract Potentially Contribute to Improving Growth, Bulb Quality and Yield of Onion (*Allium cepa*)

**DOI:** 10.3390/molecules26092633

**Published:** 2021-04-30

**Authors:** Nabil A. Younes, Md. Mezanur Rahman, Ahmed A. Wardany, Mona F. A. Dawood, Mohammad Golam Mostofa, Sanjida Sultana Keya, Arafat Abdel Hamed Abdel Latef, Lam-Son Phan Tran

**Affiliations:** 1 Horticulture Department, Faculty of Agriculture, Al-Azhar University-Assiut Branch, Assiut 71524, Egypt; nabel_aly77@yahoo.com; 2 Department of Agroforestry and Environment, Bangabandhu Sheikh Mujibur Rahman Agricultural University, Gazipur 1706, Bangladesh; mrahman@bsmrau.edu.bd (M.M.R.); sanjidakeya99@gmail.com (S.S.K.); 3 Botany and Microbiology Department, Faculty of Science, Al-Azhar University, Assiut 71524, Egypt; Ahmed_wr2000@yahoo.com; 4 Botany and Microbiology Department, Faculty of Science, Assiut University, Assiut 71516, Egypt; mo_fa87@aun.edu.eg; 5 Department of Biochemistry and Molecular Biology, Bangabandhu Sheikh Mujibur Rahman Agricultural University, Gazipur 1706, Bangladesh; mostofa@bsmrau.edu.bd; 6 Biology Department, Turabah University College, Turabah Branch, Taif University, P.O. Box 11099, Taif 21944, Saudi Arabia; 7 Institute of Research and Development, Duy Tan University, Da Nang 550000, Vietnam; 8 Department of Plant and Soil Science, Institute of Genomics for Crop Abiotic Stress Tolerance, Texas Tech University, Lubbock, TX 79409, USA

**Keywords:** antioxidants, biostimulants, bulb weight, licorice root extract, onion yield, osmoprotectants

## Abstract

The increasing culinary use of onion (*Alium cepa*) raises pressure on the current production rate, demanding sustainable approaches for increasing its productivity worldwide. Here, we aimed to investigate the beneficial effects of licorice (*Glycyrrhiza glabra*) root extract (LRE) in improving growth, yield, nutritional status, and antioxidant properties of two high-yielding onion cultivars, Shandaweel and Giza 20, growing under field conditions in two consecutive years. Our results revealed that pretreatments of both onion cultivars with LRE exhibited improved growth indices (plant height and number of leaves) and yield-related features (bulb length, bulb diameter, and bulb weight) in comparison with the corresponding LRE-devoid control plants. Pretreatments with LRE also improved the nutritional and antioxidant properties of bulbs of both cultivars, which was linked to improved mineral (e.g., K^+^ and Ca^2+^) acquisition, and heightened activities of enzymatic antioxidants (e.g., superoxide dismutase, catalase, ascorbate peroxidase, glutathione peroxidase, and glutathione *S*-transferase) and increased levels of non-enzymatic antioxidants (e.g., ascorbic acid, reduced glutathione, phenolics, and flavonoids). LRE also elevated the contents of proline, total free amino acids, total soluble carbohydrates, and water-soluble proteins in both onion bulbs. In general, both cultivars displayed positive responses to LRE pretreatments; however, the Shandaweel cultivar performed better than the Giza 20 cultivar in terms of yield and, to some extent, bulb quality. Collectively, our findings suggest that the application of LRE as biostimulant might be an effective strategy to enhance bulb quality and ultimately the productivity of onion cultivars under field conditions.

## 1. Introduction

Innovation of sustainable and environmentally friendly agricultural technologies is the biggest challenge of modern agriculture to ensure a continuous supply of food and nutrition for the burgeoning world population [1]. In addition, considering the notorious impact of climate change on crop production, depletion of arable lands, and intensification of agricultural practices, the quest for environmentally safe organic materials to achieve sustainable farming is gaining momentum. The use of biostimulants has recently been identified as a promising natural product application-based approach that offers a flexible, cost-effective, and widely applicable alternative for improving sustainable agricultural productivity while buffering the impacts of climate change [2,3]. Consequently, their extensive usage in the agricultural sector has tremendously increased, as indicated by the current global market of around USD 2.0 billion, which is projected to be expanded to USD 3.0 billion by 2021 [4].

It is well known that plant biostimulants are any substances or mixtures of substances originating from natural resources, and are distinct from nutrients and pesticides [3]. Biostimulants can stimulate plant growth, development, and stress tolerance when being applied at specific concentrations following proper guidelines [3,5]. Undeniably, the efficacy of biostimulants depends on several factors, including the standardization of their raw materials, properties, and extraction methods, as well as the identification of the optimal doses, time, and mode of application for each plant species under different growth conditions [2]. In general, biostimulants can boost crop productivity primarily by improving (i) root system architecture for enhancing uptake of water and nutrients, (ii) photosynthetic capacity for maximizing growth, and (iii) antioxidant defense system for reducing oxidative stress in plants [3,6]. Despite substantial progress in omic research on the mechanistic aspects of biostimulants in promoting plant growth and development, it is still an area of intensive research to gain information on how biostimulants are precisely involved in improving these processes. In addition, the key metabolic components of biostimulants are widely unknown; hence, their functions have not been explored either [7]. Biostimulants can be of different types depending on the sources; however, they are broadly categorized as non-microbial, microbial, or a combination of both [7]. Among natural plant-based non-microbial biostimulants, licorice (*Glycyrrhiza glabra*) root extract (LRE) is a rich source of antioxidants, osmoprotectants, and phytohormones [8,9]. Recently, it has been reported that LRE application improved growth, nutrient uptake, and yield in pea plants (*Pisum sativum*) under field conditions [10]. LRE supplementation also protected *P. sativum* and common bean plants (*Phaseolus vulgaris*) from salinity by reducing reactive oxygen species (ROS)-induced oxidative damage through the improvement of osmoprotectant levels and the antioxidant defense system [11,12]. In addition, LRE application boosted *Capsicum annum* growth and fruit yield in heavy metal-contaminated saline soils by improving the contents of photosynthetic pigments, total soluble sugars, free proline, and nutrients, as well as the activities of antioxidant enzymes in leaves [13]. Although most of the research on the use of biostimulants has focused mainly on their benefits under different stress conditions, there have been also several reports on biostimulant-induced growth stimulation when they were applied to plants grown under normal growth conditions [14,15]. In reality, in the era of dwindling agricultural lands, it should be a paramount goal of today’s plant scientists and farmers to explore biostimulants as a tonic to boost the yields of commercially valuable crops, including onions (*Allium cepa*), under field conditions.

Onion is one of the most important vegetable crops in the world, ranked second after tomato (*Solanum lycopersicum*) [16], and has been widely used as a vegetable, spice, and medicine since time immemorial [17]. Green leaves and green and/or dry onion bulbs are rich sources of vitamins, minerals, carbohydrates, proteins, and antioxidants that play a central role in protecting humans from a number of diseases, such as chickenpox, influenza, cancer, diabetes, high blood pressure, and cardiovascular disorders [17,18,19,20]. Egypt is one of the largest onion-producing countries in the world, ranked fourth in terms of exports, with a total production of 3.08 million tons from 87.95 hectares of harvested area [21]. Because onion is the leading commercial crop in Egypt, the country has therefore emphasized improving the yield and quality of onion by using the existing land area.

Considering the biostimulating effects that LRE has had on the productivity of a number of crop plants, we further explored the potential effects of LRE on enhancing onion growth and productivity under field conditions. For this purpose, we applied LRE in two different doses to two field-grown onion cultivars, namely, Shandaweel and Giza 20, and compared their performance based on various morphological, antioxidant potential, nutrient status, bulb quality, and yield-related attributes.

## 2. Results

### 2.1. Effect of LRE on the Contents of Photosynthetic Pigments and Anthocyanins in Leaves of Two Onion Cultivars

**Figure 1:**
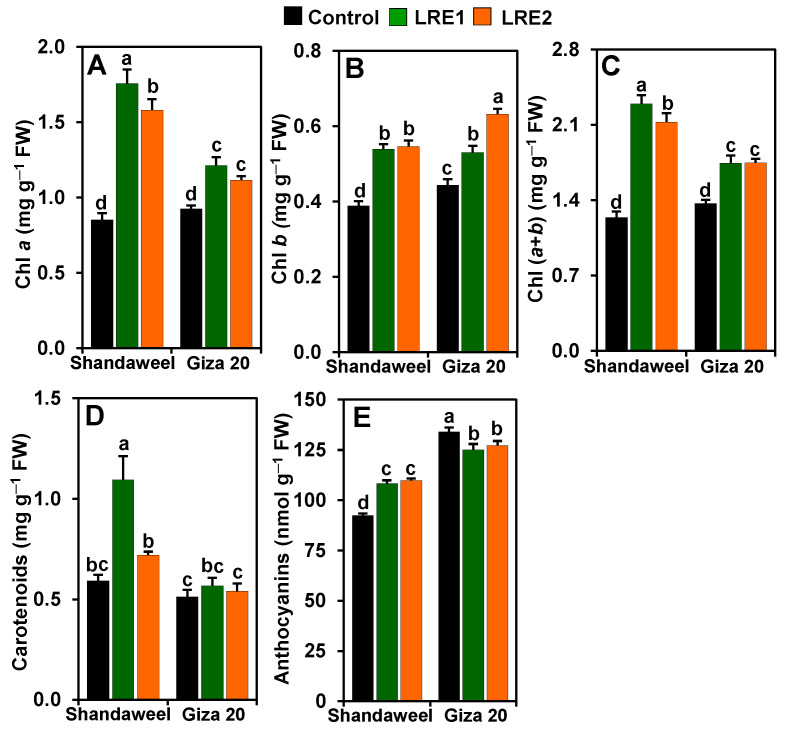
Effects of licorice root extract (LRE) on photosynthetic pigment and anthocyanin contents in the leaves of Shandaweel and Giza 20 cultivars. The contents of (**A**) chlorophyll (Chl) *a*, (**B**) Chl *b*, (**C**) Chl (*a* + *b*), (**D**) carotenoids, and (**E**) anthocyanins were measured at day 90th after transplantation. Data shown are means ± standard errors of three independent replications (*n* = 3) for each treatment. Different alphabetic letters showed statistically significant variations among treatments and cultivars according to a least significant difference test (*p* < 0.05). FW, fresh weight; LRE1, 100 g L^−1^ LRE-treated seedlings; LRE2, 200 g L^−1^ LRE-treated seedlings.

In comparison to the control, LRE1 and LRE2 Shandaweel leaves exhibited increases in the contents of chlorophyll (Chl) *a* (by 106.04 and 85.25%, respectively), Chl *b* (by 38.75 and 40.51%, respectively), and Chl (*a* + *b*) (by 84.97 and 71.24%, respectively) (Figure 1A–C). Similarly, a substantial enhancement in the contents of Chl *a* (by 31.19 and 20.71%, respectively), Chl *b* (by 19.49 and 42.45%, respectively), and Chl (*a* + *b*) (by 27.40 and 27.76%, respectively), was observed in LRE1 and LRE2 Giza 20 leaves relative to the corresponding control values (Figure 1A–C). Furthermore, although carotenoid content increased by 85.03% in LRE1 Shandaweel leaves in relation to the control, no distinct differences were observed for carotenoid contents in the control, LRE1, or LRE2 Giza 20 leaves (Figure 1D). Surprisingly, anthocyanin contents correspondingly increased by 17.23 and 18.79% in LRE1 and LRE2 Shandaweel leaves, respectively, but the contents were attenuated by 6.64 and 5.02% in LRE1 and LRE2 Giza 20 leaves, respectively, in comparison to the control (Figure 1E).

### 2.2. Effect of LRE on the Reactive Oxygen Species Levels, and Activities of Superoxide Dismutase, Catalase, Ascorbate Peroxidase, Glutathione Peroxidase, and Glutathione S-Transferase in the Bulbs of Two Onion Cultivars

**Figure 2:**
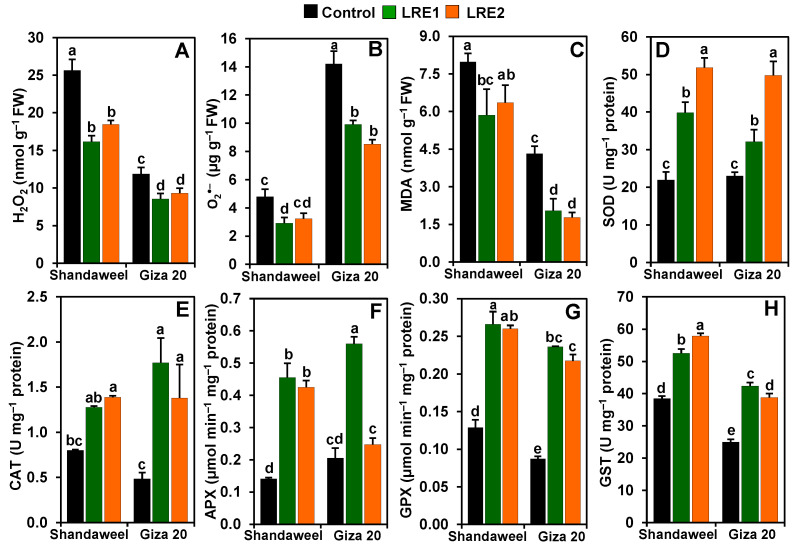
Effects of licorice root extract (LRE) on reactive oxygen species accumulation, malondialdehyde (MDA) contents, and antioxidant enzyme activities in the bulbs of Shandaweel and Giza 20 cultivars. The levels of (**A**) hydrogen peroxide (H_2_O_2_), (**B**) superoxide (O_2_^•−^), and (**C**) malondialdehyde (MDA), and the activities of (**D**) superoxide dismutase (SOD), (**E**) catalase (CAT), (**F**) ascorbate peroxidase (APX), (**G**) glutathione peroxidase (GPX), and (**H**) glutathione *S*-transferase (GST) were measured at day 90th after transplantation. Data shown are means ± standard errors of three independent replications (*n* = 3) for each treatment. Different alphabetic letters show statistically significant variations among the treatments and cultivars according to a least significant difference test (*p* < 0.05). FW, fresh weight; LRE1, 100 g L^−1^ LRE-treated seedlings; LRE2, 200 g L^−1^ LRE-treated seedlings.

Compared to the control, the levels of hydrogen peroxide (H_2_O_2_) decreased by 37.02 and 28.06% in LRE1 and LRE2 Shandaweel bulbs, respectively. Likewise, both LRE1 and LRE2 Giza 20 bulbs displayed a substantial decrease in the content of H_2_O_2_ by 27.86 and 21.46%, respectively, relative to the control (Figure 2A). Furthermore, the contents of superoxide (O_2_^•−^) and malondialdehyde (MDA) in LRE1 Shandaweel bulbs decreased by 39.01 and 26.63%, respectively (Figure 2B,C). LRE1 and LRE2 Giza 20 bulbs displayed a significant decline in the levels of O_2_^•−^ (by 30.22 and 40.08%, respectively) and MDA (52.69 and 58.96%, respectively), relative to the values of the control (Figure 2B,C). In relation to the control, a sharp enhancement in the activities of superoxide dismutase (SOD) (by 81.34 and 136.11%), ascorbate peroxidase (APX) (223.14 and 201.29%), glutathione peroxidase (GPX) (106.71 and 102.08%), and glutathione *S*-transferase (GST) (36.62 and 50.51%) was noticed in LRE1 and LRE2 Shandaweel bulbs, respectively (Figure 2D,F–H). Nevertheless, comparable levels of catalase (CAT) activity were found in the control and LRE1 Shandaweel bulbs, whereas CAT activity was enhanced by 73.29% in LRE2 Shandaweel bulbs versus the control (Figure 2E). Similarly, the activities of SOD, CAT, GPX, and GST were noticeably enhanced by 39.67, 264.64, 170.80, and 69.53% in LRE1, and by 116.35, 184.35, 149.25, and 55.24% in LRE2 Giza 20 bulbs over the corresponding control values (Figure 2D–H). Additionally, APX activity substantially increased by 172.69% in LRE1 Giza 20 bulbs relative to that of the control (Figure 2F). 

### 2.3. Effect of LRE on the Mineral Contents in the Bulbs of Two Onion Cultivars

**Figure 3:**
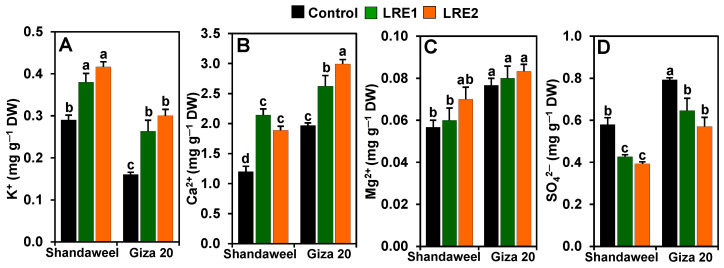
Effects of licorice root extract (LRE) on the mineral ion contents in the bulbs of Shandaweel and Giza 20 cultivars. Levels of (**A**) K^+^, (**B**) Ca^2+^, (**C**) Mg^2+^, and (**D**) SO_4_^2^^−^ were measured at day 90th after transplantation. Data shown are means ± standard errors of three independent replications (*n* = 3) for each treatment. Different alphabetic letters show statistically significant variations among the treatments and cultivars according to a least significant difference test (*p* < 0.05). DW, dry weight; LRE1, 100 g L^−1^ LRE-treated seedlings; LRE2, 200 g L^−1^ LRE-treated seedlings.

In relation to the control, the contents of K^+^ and Ca^2+^ significantly increased in LRE1 Shandaweel bulbs by 31.03 and 78.61%, respectively, and in LRE2 Shandaweel bulbs by 43.68 and 57.50%, respectively (Figure 3A,B). Likewise, a noteworthy improvement was noted for the contents of K^+^ (by 64.58 and 87.50%) and Ca^2+^ (33.16 and 51.95%) in the LRE1 and LRE2 Giza 20 bulbs, respectively, relative to the corresponding control values (Figure 3A,B). Nevertheless, LRE1 and LRE2 Shandaweel bulbs, and LRE1 and LRE2 Giza 20 bulbs displayed no significant differences in magnesium ion (Mg^2+^) levels compared to the respective control (Figure 3C). On the other hand, the contents of SO_4_^2−^ in LRE1 and LRE2 Shandaweel bulbs substantially decreased by 26.44 and 32.18%, respectively, compared to the control (Figure 3D). Similarly, LRE1 and LRE2 Giza 20 bulbs also displayed a significant decline in the content of SO_4_^2−^ (by 18.49 and 28.15%, respectively), relative to the control (Figure 3D). 

### 2.4. Effect of LRE on the Contents of Proline, Total Free Amino Acids, Total Soluble Carbohydrates, and Water-Soluble Proteins in the Bulbs of Two Onion Cultivars

**Figure 4:**
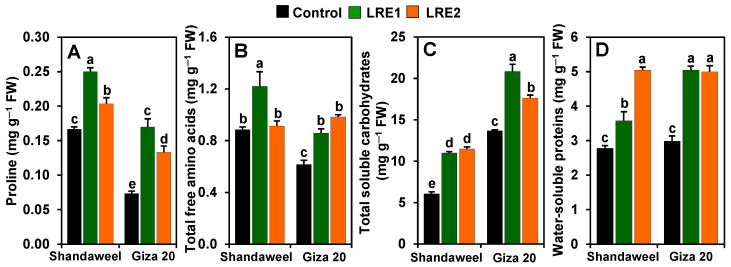
Effects of licorice root extract (LRE) on osmoprotectant accumulations in the bulbs of Shandaweel and Giza 20 cultivars. The contents of (**A**) proline, (**B**) total free amino acids, (**C**) total soluble carbohydrates, and (**D**) water-soluble proteins were measured at day 90th after transplantation. Data shown are means ± standard errors of three independent replications (*n* = 3) for each treatment. Different alphabetic letters show statistically significant variations among the treatments and cultivars according to a least significant difference test (*p* < 0.05). FW, fresh weight; LRE1, 100 g L^−1^ LRE-treated seedlings; LRE2, 200 g L^−1^ LRE-treated seedlings.

In comparison with the corresponding values obtained from the control, both LRE1 and LRE2 Shandaweel bulbs exhibited a sharp increase in the contents of proline (by 50.00 and 22.00%, respectively), total soluble carbohydrates (81.30 and 89.16%, respectively), and water-soluble proteins (28.61 and 81.20%, respectively) (Figure 4A,C,D). The total free amino acids content increased by 37.75% in LRE1 Shandaweel bulbs only in relation to the control (Figure 4B). On the other hand, the contents of proline, total free amino acids, total soluble carbohydrates, and water-soluble proteins increased by 131.82, 39.32, 52.35, and 69.04%, respectively, in LRE1, and by 81.82, 59.55, 28.92, and 67.49%, respectively, in LRE2 Giza 20 bulbs over the corresponding control values (Figure 4A–D). 

### 2.5. Effect of LRE on the Contents of Ascorbic Acid, Reduced Glutathione, Phenolics, Flavonoids, and Pyruvic Acid, and on the Total Antioxidant Capacity in the Bulbs of Two Onion Cultivars

**Figure 5:**
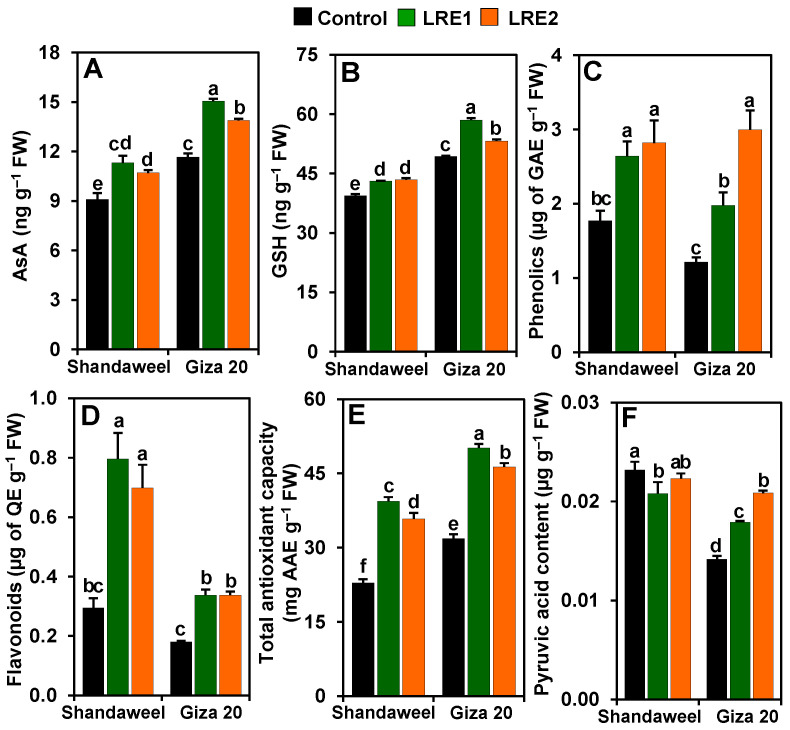
Effects of licorice root extract (LRE) on the levels of non-enzymatic antioxidants and pyruvic acid in the bulbs of Shandaweel and Giza 20 onion cultivars. The levels of (**A**) ascorbic acid (AsA), (**B**) reduced glutathione (GSH), (**C**) phenolics, (**D**) flavonoids, (**E**) total antioxidant capacity, and (**F**) pyruvic acid were measured at day 90th after transplantation. Data shown are means ± standard errors of three independent replications (*n* = 3) for each treatment. Different alphabetic letters showed statistically significant variations among the treatments and cultivars according to a least significant difference test (*p* < 0.05). AAE, L-ascorbic acid equivalent; FW, fresh weight; GAE, gallic acid equivalent; LRE1, 100 g L^−1^ LRE-treated seedlings; LRE2, 200 g L^−1^ LRE-treated seedlings; QE, quercetin equivalent.

Significant improvements in the levels of ascorbic acid (AsA) (by 24.38 and 17.67%), reduced glutathione (GSH) (9.36 and 10.24%), phenolics (49.16 and 59.31%) and flavonoids (170.25 and 137.62%), and the total antioxidant capacity (72.51 and 56.63%) were recorded in the LRE1 and LRE2 Shandaweel bulbs, respectively, when contrasted with the corresponding control values (Figure 5A–E). Nevertheless, the pyruvic acid level decreased by 10.15% in LRE1 Shandaweel bulbs only relative to the control (Figure 5F). LRE1 and LRE2 Giza 20 bulbs exhibited noteworthy improvements in the contents of AsA (by 29.01 and 19.04%, respectively), GSH (18.57 and 7.87%, respectively), phenolics (68.89 and 146.61%, respectively), flavonoids (86.93 and 86.93%, respectively), pyruvic acid (26.31 and 47.16%, respectively), and the total antioxidant capacity (57.31 and 45.40%, respectively) versus the corresponding control values (Figure 5A–F). 

### 2.6. Effect of LRE on Morphological and Yield-Contributing Attributes of Two Onion Cultivars

In the year 2016, compared to the control, LRE1 and LRE2 Shandaweel plants displayed noticeable increments in plant height (by 54.26 and 45.41%, respectively), number of leaves (31.41 and 50.42%, respectively), onion bulb length (56.16 and 51.37%, respectively), onion bulb diameter (61.49 and 59.77%, respectively), onion bulb weight (225.65 and 210.37%, respectively), and total onion yield (225.58 and 210.39%, respectively). In the same year, significant increases were also observed for plant height, onion bulb diameter, onion bulb weight, and total onion yield by 13.99, 39.44, 94.49, and 94.27%, respectively, in LRE1, and by 11.11, 27.23, 60.35, and 60.30%, respectively, in LRE2 Giza 20, compared to the control plants (Table 1). Nonetheless, in 2016, the number of leaves increased by 12.50% in LRE1 Giza 20 in relation to the control plants (Table 1). However, no significant differences were recorded between the control and the LRE1 or LRE2 Giza 20 plants for onion bulb length in 2016 (Table 1). 

**Figure 6:**
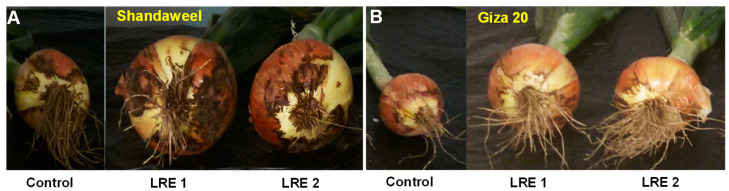
Effects of licorice root extract (LRE) on bulb growth of onion cultivars Shandaweel (**A**) and Giza 20 (**B**). The photographs were taken at day 90th after transplantation in the year 2017. LRE1, 100 g L^−1^ LRE-treated seedlings; LRE2, 200 g L^−1^ LRE-treated seedlings.

In the year 2017, both LRE1 and LRE2 Shandaweel plants showed a remarkable increase in plant height (by 59.20 and 56.36%, respectively), number of leaves (45.83 and 53.10%, respectively), onion bulb length (57.43 and 45.95%, respectively), onion bulb diameter (56.42 and 53.07%, respectively), onion bulb weight (196.69 and 186.45%, respectively), and total onion yield (209.20 and 199.01%, respectively), compared to that of the control plants (Table 1). On the other hand, in 2017, LRE1 and LRE2 Giza 20 plants displayed a substantial improvement in plant height (by 16.63 and 14.50%, respectively), number of leaves (25.50 and 18.37%, respectively), onion bulb length (46.01 and 38.65%, respectively), onion bulb diameter (31.28 and 19.82%, respectively), onion bulb weight (94.73 and 64.80%, respectively), and total onion yield (92.21 and 59.63%, respectively), in comparison to the corresponding control values (Table 1). Additionally, LRE-mediated improvements in morphological features and yield-contributing parameters of both Shandaweel and Giza 20 cultivars coincided with the better phenotypes of their bulbs when compared to the corresponding control plants (Figure 6A,B).

## 3. Discussion

Boosting onion yield with improved nutraceutical properties has received increased attention from researchers, farmers, investors, consumers, and regulators nowadays. LRE has been found to be an amazing biostimulant that increases not only growth but also the yields of various crops [2,3]. In the present study, we examined the effects of LRE on physiological and biochemical properties, oxidative marker accumulations, antioxidant activities, nutraceutical quality, and growth- and yield-related features of onion cultivars under field conditions. Undeniably, several factors determine the growth and yield of crops under field conditions, for example, crop production technology (e.g., cultivars, planting procedure, plant spacing, sowing time, fertilization, weeding, and irrigation), stresses (e.g., salinity, drought, pests, and diseases), climatic conditions (e.g., precipitation, temperature, humidity, wind speed, and solar radiation), and soil properties (e.g., temperature, moisture, aeration, organic matter, soil nutrient dynamics, and pH) [22,23,24].

It is well known that chlorophylls and carotenoids are ubiquitous and essential photosynthetic pigments, which are intricately correlated with plant biomass production [25]. In humans, carotenoids being the precursor of vitamin A also play a fundamental role in reducing the onset of some chronic diseases, such as cancers, cardiovascular diseases, and age-related eye diseases [26,27]. The results of the present study demonstrated that the use of LRE enhanced the contents of photosynthetic pigments in both onion cultivars compared to the corresponding control plants, suggesting that plants supplemented with LRE maintained better photosynthetic capacity (Figure 1A–C). Importantly, LRE-pretreated Shandaweel demonstrated greater improvement in photosynthetic pigment and anthocyanin levels than LRE-pretreated Giza 20 at both LRE concentrations (Figure 1A–C,E). In addition, the presence of Mg^2+^, a central atom in Chl pigments [28], as well as Fe^2+^ in LRE, plausibly played a decisive role in Chl biosynthesis and formation (Table 2) [12,13].

In the current study, LRE-devoid control plants of both onion cultivars exhibited a substantial accumulation of ROS, including O_2_^•−^ and H_2_O_2_, which was accompanied by a concomitant rise of MDA content in onion bulbs, indicating that the control plants might have suffered from oxidative damage under field conditions (Figure 2A–C). On the contrary, LRE-pretreated onion cultivars inhibited the accumulations of O_2_^•−^, H_2_O_2_, and MDA in onion bulbs (Figure 2A–C), suggesting that LRE played a critical role in the preparedness of both onion cultivars to fight against ROS-induced oxidative damage in onion plants (Figure 2A–C). More fundamentally, LRE pretreatments were more effective in diminishing the rise of O_2_^•−^ and MDA in Giza 20 than in Shandaweel bulbs, whereas the diminution of H_2_O_2_ was more prominent in the Shandaweel than in the Giza 20 bulbs (Figure 2A–C). Indeed, a well-balanced antioxidant protection system in plants is a prerequisite for the elimination of ROS [29,30]. The increasing trend in the activities of SOD, CAT, APX, GPX, and GST in the onion bulbs of LRE-pretreated onion cultivars also endorsed that LRE effectively provided a safeguard against this oxidative damage (Figure 2D–H). Interestingly, the onion cultivars exhibited differential levels of enzymatic activities upon treatment with LRE. For instance, the activities of CAT, GPX, and GST were higher in Giza 20 than in Shandaweel bulbs, whereas the activities of SOD and APX were higher in Shandaweel than in Giza 20 bulbs (Figure 2D–H), indicating that LRE-mediated promotion of enzyme activities is cultivar-dependent.

It is widely known that mineral nutrients present in the soil are taken up by the plant roots and eventually transported to the edible plant parts for human consumption, and they act as important co-factors for numerous enzymes involved in cellular metabolism [31]. In the present study, the results indicated that onion cultivars pretreated with LRE showed higher levels of K^+^, Ca^2+^, and Mg^2+^ in their bulbs than their LRE-free counterparts did (Figure 3A–C). With respect to the cultivars, our findings showed that both levels of LRE pretreatment significantly improved the accumulation of Ca^2+^ in the Shandaweel compared to the Giza 20 bulbs, whereas significant improvements in the accumulation of K^+^ were achieved in LRE-pretreated Giza 20 compared to the Shandaweel bulbs (Figure 3A,B). The enhancement of mineral nutrients in onion bulbs induced by LRE pretreatment may be due to the fact that LRE itself is a rich source of mineral nutrients, especially K^+^, Ca^2+^, and Mg^2+^ (Table 2), and hormones such as gibberellic acid, auxin, and zeatin-type cytokinin, which elevate several metabolic processes, including nutrient absorption, contributing to the enrichment of nutrient concentrations in plant tissues [13,32,33]. Surprisingly, we observed that the bulbs of LRE-pretreated onion cultivars displayed lower contents of SO_4_^2−^ than those of the corresponding control plants (Figure 3D), implying that LRE might contribute to the formation of various sulfur-containing compounds such as GSH (Figure 5B) under natural conditions to maintain better quality of onion cultivars [34]. It is worth noting that the content of SO_4_^2−^ was higher in the Shandaweel than in the Giza 20 bulbs following LRE pretreatment (Figure 3D).

In the current study, we also observed that LRE-pretreated onion cultivars displayed an enhanced accumulation of proline in bulbs compared to the respective control plants (Figure 4A), suggesting that LRE contributed to osmotic adjustment within plant cells and preserved the water content of the onion bulb tissues under field conditions. We also observed that the bulbs of LRE-pretreated onion cultivars demonstrated higher levels of free amino acids compared to the corresponding control plants (Figure 4B). Free amino acids are important metabolites that have been reported to transport from the senescence foliage to the onion bulbs during maturation [35]. Thus, we postulated that the observed higher numbers of leaves in LRE-pretreated onion cultivars led to a sturdier translocation of amino acids from the leaves to the onion bulbs (Table 1). Carbohydrate content, which constitutes over 80% of the dry weight of the onion bulbs [36], is another important indicator of bulb quality. The total soluble carbohydrate content in bulbs of both onion cultivars was also increased by LRE pretreatments (Figure 4C), presumably as a consequence of improved levels of photosynthetic pigments, leading to an increased photosynthetic rate (Figure 1A–C). It is also possible that LRE pretreatments might have stimulated the source-to-sink transport of sugars during the swelling phase of the onion bulbs, thereby increasing bulb carbohydrates content. In line with our results, Mazrou [37] demonstrated that LRE pretreatment enhanced the levels of total soluble carbohydrates in *Pimpinella anisum*. Furthermore, the increased water-soluble protein contents in bulbs of LRE-pretreated onion cultivars may be associated with better nitrogen assimilation and stimulation of the amino acid metabolism (Figure 4D) [38]. Intriguingly, the greater protein accumulation in bulbs of LRE-pretreated onion cultivars suggests that these bulbs would supply sufficient proteins for multiple body functions in consumers. It is important to note that LRE pretreatments were more effective in stimulating the levels of proline, total free amino acids, and water-soluble proteins in Giza 20 than in Shandaweel, yet were more effective at enhancing total soluble carbohydrate content in Shandaweel than in Giza 20 bulbs (Figure 4A–D).

We further elucidated the beneficial role of LRE by quantifying the amount of non-enzymatic antioxidants in onion bulbs. Our data demonstrated that the pretreatments of onion cultivars with LRE elevated the concentrations of AsA, GSH, phenolics, and flavonoids, as well as the total antioxidant capacity, relative to their levels in the corresponding control plants (Figure 5A–E). With respect to the cultivars, our findings indicated that both levels of LRE pretreatment resulted in increased contents of AsA, GSH, and phenolics in the Giza 20 compared to the Shandaweel bulbs, whereas the flavonoid levels and the total antioxidant capacity were better in Shandaweel than in Giza 20 bulbs (Figure 5A–E). Our results suggest that LRE-pretreated onion cultivars absorbed antioxidants such as AsA, phenolics, and flavonoids that are present in LRE (Table 2) [13,38], and subsequently increased their endogenous levels to enable onion cultivars to counteract the ROS-induced oxidative damage. Intriguingly, these antioxidants also perform the most fundamental functions in the human body. For examples, AsA and GSH are imperative for maintaining the functions of collagen, carnitine, and neurotransmitters, as well as preventing various life-threatening diseases like Parkinson’s and liver disease in the human body [39,40]. Phenolics and flavonoids have received increasing medicinal importance owing to their enormous dietary health benefits and functionalities, such as antioxidant, immuno-regulatory, anti-inflammatory, anti-atherogenic, anti-allergic, anti-thrombotic, anti-microbial, cardio protective, and anti-cancer activities and anti-diabetic properties [41,42]. Because onion bulbs serve as a common ingredient in various foodstuffs, the intake of onion bulbs rich in antioxidants may improve the ability of the human body to scavenge free radicals to avoid their harmful effects on vital biomolecules and ultimately body tissues [43].

Pungency level is an important quality attribute in onion bulb. Although onion pungency is preferable in many applications (e.g., sauce), low-pungent onion cultivars are highly preferable in households, as well as in extensive use in retail, food service, and numerous food industries, owing to their minimal lachrymatory properties and ease of use as a fresh and uncooked ingredient [44,45]. Pyruvic acid, a product accompanied by the formation of different thiosulfinates through alliinase, is widely recognized as a measure of the pungency index in onions [46]. Our findings displayed that the pretreatments with LRE kept the pyruvic acid content unchanged in the bulb of the Shandaweel cultivar, whereas it dramatically increased it in the Giza 20 cultivar (Figure 5F), indicating that LRE actions on pungency might be cultivar-dependent. Thus, LRE pretreatment could be used for suppression of pungency in the Shandaweel cultivar for better acceptability by end-users.

Apart from the quality attributes, we also focused on the growth and yield-related features of onion cultivars to confirm the beneficiary roles of the LRE application. Our results showed that LRE pretreatments also enhanced growth indices, such as plant height and the number of leaves (Table 1), which coincided with the increase in photosynthetic pigments (Figure 1A–C), thereby leading to enhanced onion bulb length, onion bulb diameter, and onion bulb weight, as the photoassimilates produced in the above-ground part follow the source-to-sink transition during the maturation phase of the onion bulb (Figure 6A,B) [47]. Our results also clearly revealed that the morphological and yield-contributing features of the Shandaweel cultivar were more positively modulated by LRE pretreatments in both years compared to those of the Giza 20 plants (Table 1). 

## 4. Materials and Methods

### 4.1. Study Area, Climatic Conditions, Soil Properties, and Plant Materials

The field experiments were performed at the research farm of the Faculty of Agriculture, Al-Azhar University, Assiut, Egypt (longitude 31°11′21.42″ E and latitude 27°10′48.48″ N) from November to April for two consecutive years in 2016 and 2017. The study site is characterized by a sub-tropical climate with hot summer and mild winter. The temperatures of the research area fluctuated from 10 to 28 °C in 2016 and 11 to 27 °C in 2017. Prior to field experimentation, soil samples were collected at a depth of 0 to 30 cm in order to determine various physical and chemical properties following the comprehensive protocols described in Carter and Gregorich [48], and the results are summarized in Table S1. In the present study, two onion cultivars, including Shandaweel and Giza 20, were used to evaluate the efficacy of LRE in improving morphological, physiological, biochemical, and yield-contributing features. These two cultivars are extensively cultivated throughout the country as they have high yield potential and are widely accepted by both producers and consumers [49].

### 4.2. Biostimulant Preparation

The commercial LRE (Sekem Group, Cairo, Egypt) was mixed in water at concentrations of 100 and 200 g L^−1^ (presented as LRE1 and LRE2, respectively). After keeping at room temperature for 24 h, the solutions were thoroughly mixed using a blender followed by filtration through filter papers (Whatman No. 42) to obtain the condensed brown liquid extracts. Gelatin (Sigma-Aldrich) (2 g L^−1^) was added to the filtered solutions of both LRE1 and LRE2 following the methodology of Younes et al. [50]. The resultant mixture was then heated at 30 ± 2 °C with stirring until properly mixed and stored for the treatments of onion bulbs. Importantly, the chemical composition of LRE was also analyzed, and several chemical constituents are shown in Table 2. Briefly, the contents of several mineral ions, for example, Na^+,^ K^+^, Ca^2+^, Mg^2+^, Fe^2+^, and Zn^2+^, were quantified using an atomic absorption spectrophotometer (Shimadzu, AA-630-02, Kyoto, Japan) following the method reported in Williams and Twine [51]. Total phenol and total flavonoid contents were assessed according to the procedures of Aery [52] and Zou et al. [53], respectively. The method of Prieto et al. [54] was followed for the determination of total antioxidant capacity. Tannin and saponin contents were quantified following the procedures of Broadhurst and Jones [55] and Hiai et al. [56], respectively. AsA content was measured according to the published method [57]. Ferric reducing antioxidant power was assessed using the method of Oyaizu [58].

### 4.3. Field Preparation and Experimental Layout

Prior to seed sowing, seeds of each cultivar were treated with Vitavax (5 g kg^−1^) to avoid spoilage from diseases. Subsequently, the treated seeds of each cultivar were sown in nursery beds with a size of 3 m × 1.2 m × 10–15 cm (length × wide × height) in order to raise seedlings. The seedbed soils were drenched with chlorpyriphos at 2 mL L^−1^ to kill soil-born insect pests. Nevertheless, prior to transplanting, plowing was done 4–5 times to obtain a good tilth followed by laddering three times for proper labeling. Nitrogen fertilizer in the form of ammonium sulfate (21%) was applied at the rate of 120 kg ha^−1^, of which one-third was applied one week before transplanting the onion seedlings as a basal dose, one-third at the early vegetative phase [30 days after transplanting (DAT)], and the last third applied 60 days after the previous one. Other basal fertilizer applications were 30 kg phosphorus (P) ha^−1^ in the form of triple superphosphate (TSP), 80 kg potassium (K) ha^−1^ in the form of potassium sulfate, and 110 kg gypsum ha^−1^. Furthermore, 25 tons of farmyard manure per hectare were also applied during land preparation.

**Figure 7:**
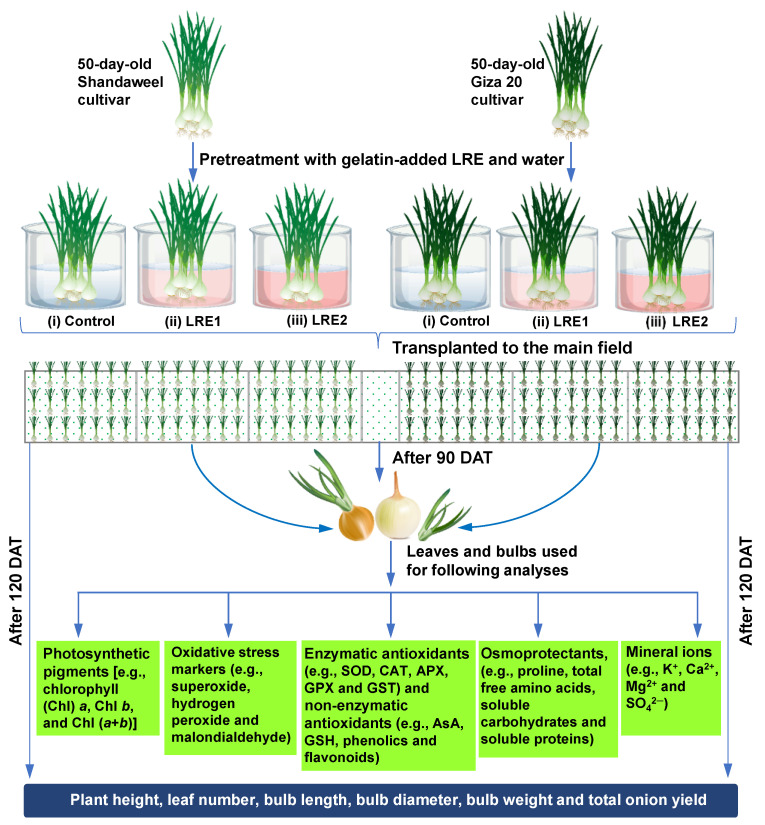
Fifty-day-old onion bulbs of Shandaweel and Giza 20 cultivars were coated with LRE1 and LRE2 by immersing them in gelatin-added LRE1 and LRE2 solutions for a period of 30 min. The control seedlings of each cultivar were coated with LRE-free gelatin solution only. For each cultivar, the experiment comprised three treatments, including (**i**) control (gelatin-treated seedlings), (**ii**) LRE1 (100 g L^−1^ LRE-treated seedlings), and (**iii**) LRE2 (200 g L^−1^ LRE-treated seedlings). Seedlings were transplanted to the main field after pretreatment. Following 90 days after transplanting (DAT), leaf and bulb samples were collected from each treatment composition to analyze different biochemical parameters. After 120 DAT, plant height and yield-associated features were recorded from the different treatment compositions of each cultivar. APX, ascorbate peroxidase; AsA, ascorbic acid; CAT, catalase; GPX, glutathione peroxidase; GSH, reduced glutathione; GST, glutathione *S*-transferase; LRE, licorice root extract; SOD, superoxide dismutase.

Before transplantation to the field, 50-day-old onion bulbs of both cultivars were coated with LRE1 and LRE2 by immersing them in gelatin-added LRE1 and LRE2 solutions for a period of 30 min. The control seedlings of each cultivar were coated with LRE-free gelatin solution. The randomized complete block design with three replicates with an area of 10.5 m^2^ (3.5 m × 3 m) for each plot was used in this study. Notably, the seedlings of each cultivar were transplanted on ridges at a spacing of 45 cm between two ridges and 10 cm from plant to plant. Light irrigation once a week was applied for optimal bulb development and boost in yield and quality of the produce. It is worth noting that the irrigation was completely stopped prior to 20 days of harvesting to dry up the outer scale and enhance the shelf life of the onion. Additionally, weeding was done regularly to keep the field free from weed infestation. Nonetheless, at 90 DAT in the year 2016, leaf and bulb samples of each cultivar were collected according to different treatment combinations for quantification of the various physiological and biochemical attributes. A schematic representation of the total experimental outline is presented in Figure 7.

### 4.4. Quantification of Photosynthetic Pigment and Anthocyanin Contents

The contents of photosynthetic pigments, including Chl *a*, Chl *b*, Chl (*a* + *b*), and carotenoids, in the leaf samples of each onion cultivar under different treatment conditions were quantified according to the method proposed by Lichtenthaler and Wellburn [59]. Anthocyanin levels in the leaves of both onion cultivars were determined according to the method of Krizek et al. [60].

### 4.5. Estimation of Mineral Nutrient Contents

The onion bulb samples (0.1 g) were oven-dried for 15 days at 60 °C and ground using mortars and pestles. The ground samples were digested with 5 mL of HNO_3_:HClO_4_ (5:1; *v*/*v*) on a hot plate at 190 °C for 2 h. The digested and subsequently filtered solutions were then used to analyze the contents of K^+^, Ca^2+^, and Mg^2+^ using a flame photometer (Carl-Zeiss DR LANGE M7D model) according to the method of Williams and Twine [51]. The turbidimetric method was followed for the determination of SO_4_^2−^ content in the water extract of dried onion bulbs following the procedure of Bardsley and Lancaster [61]. 

### 4.6. Determination of O_2_^•−^, H_2_O_2_, and MDA Contents

The levels of O_2_^•−^ and H_2_O_2_ in onion bulbs were determined according to the methods previously reported by Yang et al. [62] and Yu et al. [63], respectively. MDA content in onion bulb tissues was quantified following the procedure of Heath and Packer [64].

### 4.7. Extraction of Enzymes and Assessment of Enzyme Activities

Enzyme extracts were prepared from onion bulb tissues following the procedure described by Dawood and Azooz [65]. Determination of the activities of SOD (EC: 1.15.1.1), CAT (EC: 1.11.1.6), APX (EC: 1.11.1.11), GPX (EC: 1.11.1.9), and GST (EC: 2.5.1.18) were carried out according to the methods of Misra and Fridovich [66], Noctor et al. [67], Silva et al. [68], Flohé and Günzler [69], and Ghelfi et al. [70], respectively. Additionally, the total antioxidant capacity in the onion bulbs was determined following the protocol described by Prieto et al. [54].

### 4.8. Estimation of the Contents of Proline, Total Free Amino Acids, Water-Soluble Proteins, and Total Soluble Carbohydrates

The contents of proline, total free amino acids, water-soluble proteins, and total soluble carbohydrates in onion bulbs were determined according to the methods of Zhang and Huang [71], Lee and Takahashi [72], Bradford [73], and Ci et al. [74], respectively. 

### 4.9. Determination of the Contents of AsA, GSH, Phenolics, Flavonoids, and Pyruvic Acid 

The contents of AsA and GSH in onion bulbs were estimated following the methods described in Jagota and Dani [75] and Ellman [76], respectively. The contents of phenolics and flavonoids in onion bulbs were estimated according to the methods of Aery [52] and Zou et al. [53], respectively. Total pyruvic acid content in onion bulbs was determined according to the procedure reported by Randle and Bussard [77]. 

### 4.10. Morphological and Yield-Contributing Attributes 

Individual plant height and number of leaves in both onion cultivars were measured using a measuring scale and the simple counting method, respectively, at 120 DAT. After harvesting the onion bulbs, the bulb length and bulb diameter were immediately measured using a slide caliper. Furthermore, individual bulb weight was determined using digital balance, and subsequently, the total fresh yield of onion bulbs was computed and expressed as ton ha^−1^. 

### 4.11. Statistical Analysis

The data used to produce the figures and tables are means ± standard errors of three independent replications of each treatment. Data analysis was performed using a two-way analysis of variance (ANOVA) followed by a post hoc test, namely, the least significant difference test, to evaluate the interactions between the two factors, namely, variety and biostimulant dosage, and to compare the treatment means at *p* < 0.05. All statistical tests were done using the Statistix software (version 10.0).

## 5. Conclusions

Any changes affecting the recommended cultivation technology, as well as the surrounding climate under field conditions, may undermine the optimum crop growth and yield, which could be attributed to decreased photosynthetic capacity and increased generation of ROS. The LRE pretreatments, especially at the level of 100 g L^−1^, could reverse this trend, indicating that LRE could play a critical role in promoting onion growth, and consequently yield, and in protecting onion cultivars from oxidative damage. Furthermore, LRE pretreatments significantly improved the yield-associated characteristics and nutraceutical properties of the bulbs of both onion cultivars, demonstrating the boosting effect of LRE in improving onion quality under natural conditions. 

## Figures and Tables

**Table 1:** Effects of licorice root extract (LRE) on the growth and yield-contributing attributes of Shandaweel and Giza 20 onion cultivars at day 120th after transplantation.

Varieties	Treatments	Plant Height (cm)		Leaf Number		Bulb Length (cm)	
		2016	2017	2016	2017	2016	2017
Shandaweel	Control	49.92 ± 0.93 ^d^	48.89 ± 0.78 ^d^	10.08 ± 0.08 ^d^	9.94 ± 0.14 ^c^	4.87 ± 0.44 ^b^	4.93 ± 0.15 ^d^
	LRE1	77.00 ± 2.29 ^a^	77.83 ± 0.58 ^a^	13.25 ± 0.58 ^bc^	14.50 ± 0.54 ^ab^	7.60 ± 0.29 ^a^	7.77 ± 0.09 ^a^
	LRE2	72.58 ± 2.73 ^ab^	76.44 ± 0.48 ^a^	15.17 ± 0.58 ^a^	15.22 ± 0.55 ^a^	7.37 ± 0.12 ^a^	7.20 ± 0.15 ^b^
Giza 20	Control	60.75 ± 1.50 ^c^	59.78 ± 1.73 ^c^	12.00 ± 0.50 ^c^	10.89 ± 0.91 ^c^	7.53 ± 0.27 ^a^	5.43 ± 0.23 ^c^
	LRE1	69.25 ± 0.25 ^b^	69.72 ± 0.79 ^b^	13.50 ± 0.50 ^b^	13.67 ± 0.51 ^ab^	7.90 ± 0.06 ^a^	7.93 ± 0.03 ^a^
	LRE2	67.50 ± 2.02 ^b^	68.44 ± 0.69 ^b^	12.33 ± 0.22 ^bc^	12.89 ± 0.22 ^b^	7.60 ± 0.12 ^a^	7.53 ± 0.09 ^ab^
**Varieties**	**Treatments**	**Bulb Diameter (cm)**		**Bulb Weight (g)**		**Total Onion Yield (ton ha^−1^)**	
		**2016**	**2017**	**2016**	**2017**	**2016**	**2017**
Shandaweel	Control	5.80 ± 0.23 ^c^	5.97 ± 0.38 ^d^	115.67 ± 17.63 ^b^	130.33 ± 15.39 ^d^	29.76 ± 4.54 ^b^	31.16 ± 1.28 ^e^
	LRE1	9.37 ± 0.35 ^a^	9.33 ± 0.23 ^ab^	376.67 ± 36.89 ^a^	386.67 ± 36.71 ^a^	96.90 ± 9.46 ^a^	96.34 ± 1.44 ^a^
	LRE2	9.27 ± 0.41 ^a^	9.13 ± 0.23 ^b^	359.00 ± 29.74 ^a^	373.33 ± 8.41 ^ab^	92.38 ± 7.63 ^a^	93.16 ± 1.52 ^b^
Giza 20	Control	7.10 ± 0.31 ^b^	7.57 ± 0.20 ^c^	193.33 ± 23.95 ^b^	196.00 ± 3.06 ^c^	49.76 ± 6.12 ^b^	50.52 ± 0.41 ^d^
	LRE1	9.90 ± 0.12 ^a^	9.93 ± 0.07 ^a^	376.00 ± 22.54 ^a^	381.67 ± 2.73 ^a^	96.67 ± 5.78 ^a^	97.11 ± 0.92 ^a^
	LRE2	9.03 ± 0.33 ^a^	9.07 ± 0.09 ^b^	310.00 ± 23.39 ^a^	323.00 ± 5.03 ^b^	79.76 ± 5.98 ^a^	80.65 ± 1.38 ^c^

Data shown are means ± standard errors of three independent replications (*n* = 3) for each treatment. Different alphabetic letters showed statistically significant variations among the treatments and cultivars according to a least significant difference test (*p* < 0.05). LRE1, 100 g L^−1^ LRE-treated seedlings; LRE2, 200 g L^−1^ LRE-treated seedlings. ha, hectare.

**Table 2:** Several chemical constituents of licorice root extract (LRE).

Constituents	Values
Na^+^ (mg 100 mg^−1^ DW)	101.54
K^+^ (mg 100 mg^−1^ DW)	376.96
Ca^2+^ (mg 100 mg^−1^ DW)	754.90
Mg^2+^ (mg 100 mg^−1^ DW)	524.78
Fe^2+^ (mg 100 mg^−1^ DW)	32.88
Zn^2+^ (mg 100 mg^−1^ DW)	0.91
Total phenols (mg of GAE g^−1^ DW)	4.38
Total flavonoids (mg of QE g^−1^ DW)	2.14
Total antioxidant capacity (mg AAE g^−1^ DW)	72.99
Tannins (mg 100 mg^−1^ DW)	33.98
Saponins (mg 100 mg^−1^ DW)	21.00
Ascorbic acid (mg 100 mg^−1^ DW)	2.77
Ferric reducing antioxidant power (mg 100 mg^−1^ DW)	43.90

AAE, L-ascorbic acid equivalent; DW, dry weight; GAE, gallic acid equivalent; QE, quercetin equivalent; mg, milligram.

## Data Availability

All data included in this study are available upon request by contact with the corresponding author.

## Supplementary Materials

The following are available online. Table S1: The mean physical and chemical properties of the soil of the experimental site.

## Sample Availability

Samples of the compounds for this experiment are available upon request by contact with the corresponding author.
