# Supplementary material for: Antioxidants and Bioactive Compounds in Licorice Root Extract Potentially Contribute to Improving Growth, Bulb Quality and Yield of Onion (Allium cepa)
Source: Molecules. 2021 Apr 30;26(9):2633. doi: 10.3390/molecules26092633 (PMC8124151; doi:10.3390/molecules26092633)
Supplement: Supplementary file 1 [file molecules-26-02633-s001.zip › molecules-1176635-supplementary.pdf]

Antioxidants and Bioactive Compounds in Licorice Root Extract Potentially Contribute to Improving Growth, Bulb Quality and Yield of Onion (*Allium cepa*)

Supplementary Material

Table S1. The mean physical and chemical properties of the soil of the experimental site.

<table>
<tr><td rowspan="2">Physical properties</td><td colspan="3">Sand (%)</td><td colspan="4">Silt (%)</td><td colspan="2">Clay (%)</td><td colspan="5">Texture class</td></tr>
<tr><td colspan="3">25.5</td><td colspan="4">39.8</td><td colspan="2">34.7</td><td colspan="5">Clay loam</td></tr>
<tr><td rowspan="3">Chemical properties</td><td rowspan="2">EC (dS m^{-1})</td><td rowspan="2">pH</td><td rowspan="2">OM (%)</td><td rowspan="2">$CaCO_3$ (%)</td><td colspan="5">Soluble ions (meq L^{-1})</td><td colspan="5">Available (ppm)</td></tr>
<tr><td>HCO_3^-</td><td>Cl^-</td><td>Mg^{2+}</td><td>Na^+</td><td>K^+</td><td>N</td><td>P</td><td>K^+</td><td>SO_4^{2-}</td><td>Ca^{2+}</td></tr>
<tr><td>1.2</td><td>7.6</td><td>1.2</td><td>2.7</td><td>2.41</td><td>2.2</td><td>1.9</td><td>6.2</td><td>0.21</td><td>62.4</td><td>9.2</td><td>356</td><td>6.6</td><td>4.3</td></tr>
</table>

Abbreviations: EC, electrical conductivity; meq L^{-1}, milliequivalent per liter; OM, organic matter.
